# Achieving proportional representation in a reproductive health survey through social media: process and recommendations

**DOI:** 10.1186/s12889-022-13774-w

**Published:** 2022-07-17

**Authors:** Ona L. McCarthy, Melissa J. Palmer, Anasztazia Gubijev, Kaye Wellings, Sue Mann, Lydia Leon, Faye Callaghan, Sophie Patterson, Rebecca S. French

**Affiliations:** 1 London School of Hygiene and Tropical Medicine, London, UK; 2 Public Health England, London, UK; 3 Faculty of Health and Medicine, Lancaster University, Lancaster, UK

**Keywords:** Reproductive health, Internet survey, Web survey, Online survey, Survey methods

## Abstract

**Background:**

The narrative surrounding women’s reproductive health has shifted from a medical model to an emphasis on reproductive well-being over different life-stages. We developed and piloted a tracker survey for monitoring women’s reproductive health and well-being in England, recruiting respondents online. This paper reports on the success of the online recruitment strategies in achieving a sample proportionally representative of the England general population.

**Methods:**

Recruitment was through Facebook and Instagram advertisements and dissemination through Twitter and a blog. At the end week one, the sample was reviewed and compared to the 2011 Census England population. From week two, recruitment targeted under-represented groups. Key data were compared with prevalence estimates from the Third National Survey of Sexual Attitudes and Lifestyles (Natsal-3).

**Results:**

Between 1 July-17 August 2021, 13,962 people initiated the online survey, with 11,578 completing it. Numbers were low initially, but peaked at 1700 survey initiations per day after increasing the daily advertisement budget on day seven. At the end of week one, minority ethnic groups and people without a degree or equivalent were under-represented. From week two, we altered the advertisement settings to show to people whose profile indicated they were a ‘high school leaver’ had ‘up to some high school’, worked in industries that do not typically require a degree or lived in local authorities with a high proportion of ethnic minority residents. This had a modest effect, with the final sample short of proportional representation in terms of ethnicity and education but close in terms of region and age. Compared to Natsal-3, we found consistency in the proportion of respondents reporting an abortion and a live birth in the last year, however, the proportion of our sample reporting ever having experienced infertility was significantly higher than in Natsal-3, as was the proportion of ‘planned’ pregnancies in the last year.

**Conclusions:**

It is possible to recruit large numbers of respondents online, relatively quickly, to complete a reproductive health survey. This will be valuable to track reproductive health and well-being at a national level over time. More work is needed to understand reasons for non-response among under-represented groups.

## Background

In women the reproductive stage starts at menarche and ends at menopause. The mean age of menarche in the UK is 12.3 years [1] and for natural menopause is around 50 years [2, 3]. In between, other events or conditions may affect women’s reproductive health status, including pregnancy, infertility and gynaecological problems. These are often inter-related and the reproductive life stage will be affected by social factors as well as biological ones [4]. The World Health Organisation’s definition of reproductive health does not just focus on physical well-being but encompasses mental and social well-being, sexual satisfaction, choice and capacity [5, 6]. However, to date our knowledge and understanding of reproductive well-being and quality of life amongst women in England (including their reproductive experiences and access to knowledge, support and services) is lacking.

In 2018, Public Health England (PHE) published a series of documents [7] that emphasised reproductive health as a population and public health issue, with a life-stage and wellness approach. This shift in the narrative in England from placing emphasis on the absence of disease to reproductive wellness, together with current government policy initiatives (e.g. the rolling out of statutory Relationships and Sex Education for secondary pupils from September 2020 and placing health inequalities as a central theme in the Women’s Health Strategy) signalled the importance of tracking women’s reproductive health, well-being and experiences among the diversity of women in England through their life-stages.

The National Survey of Sexual Attitudes and Lifestyles (Natsal) is a cross-sectional probability sample survey of residents in Britain and one of the largest and most rigorous sexual behaviour surveys in the world [8]. It has been repeated approximately every decade since first conducted in 1989. As the most comprehensive source of data on reproductive health in Britain, the survey captures key variables across the reproductive life-stages, including periods; onset of sexual activity, contraception; fertility intentions and infertility; pregnancy history; family formation and menopause. However, due to constraints on what can be asked in Natsal, some of these questions are not asked in-depth and some of the priority reproductive health areas relating to well-being and quality of life are not included, such as period poverty, experience of contraceptive side effects and satisfaction with reproductive health services.

Online non-probability surveys are increasingly used, including in sexual and reproductive health research [9, 10]. In 2018 PHE also conducted a digital survey, which recruited 7500 women in England, indicating proof of concept [11]. Conducting surveys online can be efficient and convenient; in the United Kingdom (UK), 92% of all adults reported recent Internet use in 2020, which was almost universal among 16–44 year olds at 99% [12]. Social media is used by nearly three-quarters of the UK population; with Facebook being the most commonly used at 60% of all social media users, followed by WhatsApp (59%), Instagram (33%) and Twitter (25%) [13]. Online non-probability surveys distributed via social media provide a cheaper and faster alternative to probability surveys, such as Natsal [14]. However, concerns have been raised about low recruitment accrual and reach, differing user profiles by type of social media and the lack of representativeness of users and/or responders [15], for example having a social media profile declines with age and with lower socio-economic status [16].

### Background of the survey development

In 2020, PHE commissioned researchers at the London School of Hygiene and Tropical Medicine to develop and pilot a women’s reproductive health tracker survey, to be administered online. The online strategy was chosen so that it could be used in both academic and non-academic settings, in local areas and for its potential to serve as a regular national barometer of reproductive health, without incurring the costs associated with achieving a proportionally representative national sample. We sought self-reported data to help fill the void in understanding reproductive health experiences, that routine service use and pregnancy outcome data cannot fill.

People eligible to complete the survey were women who live in England aged 16–55 years. It was also inclusive of, while not specifically targeted at, those who were described as female at birth, but identified as trans male or non-binary. The aim was to develop a survey to regularly track a comprehensive suite of reproductive health variables, the data from which would be useable by colleagues working in academia, reproductive health policy and service delivery.

To develop the survey, we first created a matrix of reproductive health stages and thematic concepts relating to the fulfilment of reproductive intentions, supporting reproductive wellness and identification of reproductive morbidities. We mapped questions developed for Natsal-4 onto the matrix to identify any thematic gaps. To fill the gaps, we conducted a rapid literature review for existing validated surveys. We then reviewed the surveys and identified relevant and appropriate items to cover the themes of the survey that were not included by Natsal and also developed our own question items, guided by Natsal question and response structure. We conducted eight cognitive interviews with community-based volunteers (purposively selected to represent a range of demographics and reproductive health experiences) who completed the draft version of the survey. The cognitive interviews, feedback from members of The Royal College of Obstetrician and Gynaecologist’s Women’s Network, our wider network of academics who specialise in sexual and reproductive health and survey development and core team internal iterative testing led to the final set of survey questions.

The survey consists of 108 questions, which include those on demographics and health and covers four reproductive health themes: menstrual health, reproductive intentions, reproductive experience and reproductive ill-health. Many questions are routed so that respondents see questions that are relevant to their experience, based on their earlier question responses. Forty-five of the 108 questions (42%) were ‘new’ i.e. generated by the team and not closely based on Natsal or another survey. New questions and Natsal questions were included within all themes. Occasionally, Natsal questions were adapted in ways that maintained their comparability to the original question (for example, adding additional response items while maintaining the original items). Twenty questions were from or based on questions included in surveys identified in the rapid review. Snap Surveys, an online survey platform, was used to collect and manage the data.

The aim of this paper is to report the success of the social media recruitment strategy in producing a proportionally representative sample in this online non-probability survey compared to key demographics amongst women in England, to inform future waves of the survey.

## Methods

### Social media recruitment strategies

Specific objectives of the pilot recruitment strategy were to:

1. achieve a sample broadly reflective of the population, in terms of age, ethnicity, education level and region
2. enable testable assumptions regarding the success of the advertisements and social media platforms used for recruitment to the survey

This pilot survey was not conducted to generate prevalence estimates of reproductive health outcomes, so we did not have a target sample size. We used social media to recruit participants, resulting in a non-probability convenience sample. We planned to conduct the online recruitment in multiple, overlapping phases, allowing for strategy adaptation over the course of survey implementation to respond to the success and challenges learned from daily monitoring of survey respondent numbers and demographics. We did not use quota sampling because it would be complicated to restrict the online survey to specific groups if they had seen and clicked on a survey advertisement, there were no drawbacks to having some groups over-represented and no financial reason to restrict completion because there were no incentives for taking part.

We carried out survey recruitment through Facebook and Instagram using eight paid-for advertisements consisting of four stock images. Images were selected for inclusion of women of different ages and ethnicities and had been reviewed by and found acceptable to the Patient and Public Involvement group members during consultations at the development phase. We chose to start with these social media platforms for their high penetration in England and varied user demographics. During this phase, we set the advertisements to show to Facebook and Instagram users whose profile indicated that they lived in England, were female and were aged 16–55 inclusive. This initial approach to recruitment would provide information on how successful the advertisements were alone, at achieving a broadly proportionally representative sample, without targeting specific groups.

Towards the end of the first week, we reviewed the sample geographic and demographic spread, which we compared to England’s regional demographics using postcode data collected in the survey. Once we felt confident that we understood the success of the non-targeted minimal approach, we adjusted the advertisement targeting and images, in an attempt to correct for any under-represented groups, where possible. For example, for education level, we restricted the advertisements to people who indicated having ‘up to some high school’, ‘high school leaver’ or to people that work in an industry that does not typically require a university degree, such as work in catering and retail-oriented jobs. [The Facebook ad manager no longer enables direct advertisement targeting by ethnic group, because it was revealed to have been used in the past as vehicle for discriminatory housing advertisements.]

After the first week that the survey was live, PHE promoted it through their media channels- a blog published on their website and two Twitter tweets – and LSHTM also tweeted. We also worked with the Runnymede Trust (a UK independent race equality think tank) and Race Equality Foundation to promote the survey among ethnic minority communities, who tweeted about the study. We did not actively promote the survey to sexual and reproductive health focused organisations to avoid an over-representation of women with experience of reproductive health issues.

### Analysis

We present the number of survey initiations by day since survey launch alongside key efforts made during the recruitment period. We also present the demographic profile (age, ethnicity, highest educational level, region of residence) of the sample achieved in each week of recruitment, and the characteristics of the final sample alongside the relevant figures from the 2011 Census.

We calculated the proportion of respondents reporting key reproductive health outcomes and compared this to the corresponding prevalence estimates from Natsal-3 data (restricted to women resident in England aged 16–55), and government statistics where applicable (abortion in the last year and live birth in the last year). The health outcomes included a measure of infertility (based on asking respondents whether they had ever had a period of 12 months during which they were trying to get pregnant and this did not happen), a measure of menopausal status (defined as not having had a period for at least a year among women aged 45 or over), and the London Measure of Unplanned Pregnancy (LMUP) which is a validated six item measure to assess to what extent a pregnancy in the last year was planned [17, 18]. As part of this comparison, we also present the outcome proportions calculated after having weighted the survey data to match the 2011 Census population in terms of age and region of residence.

## Results

### Success of the social media recruitment strategies

**Fig. 1:**
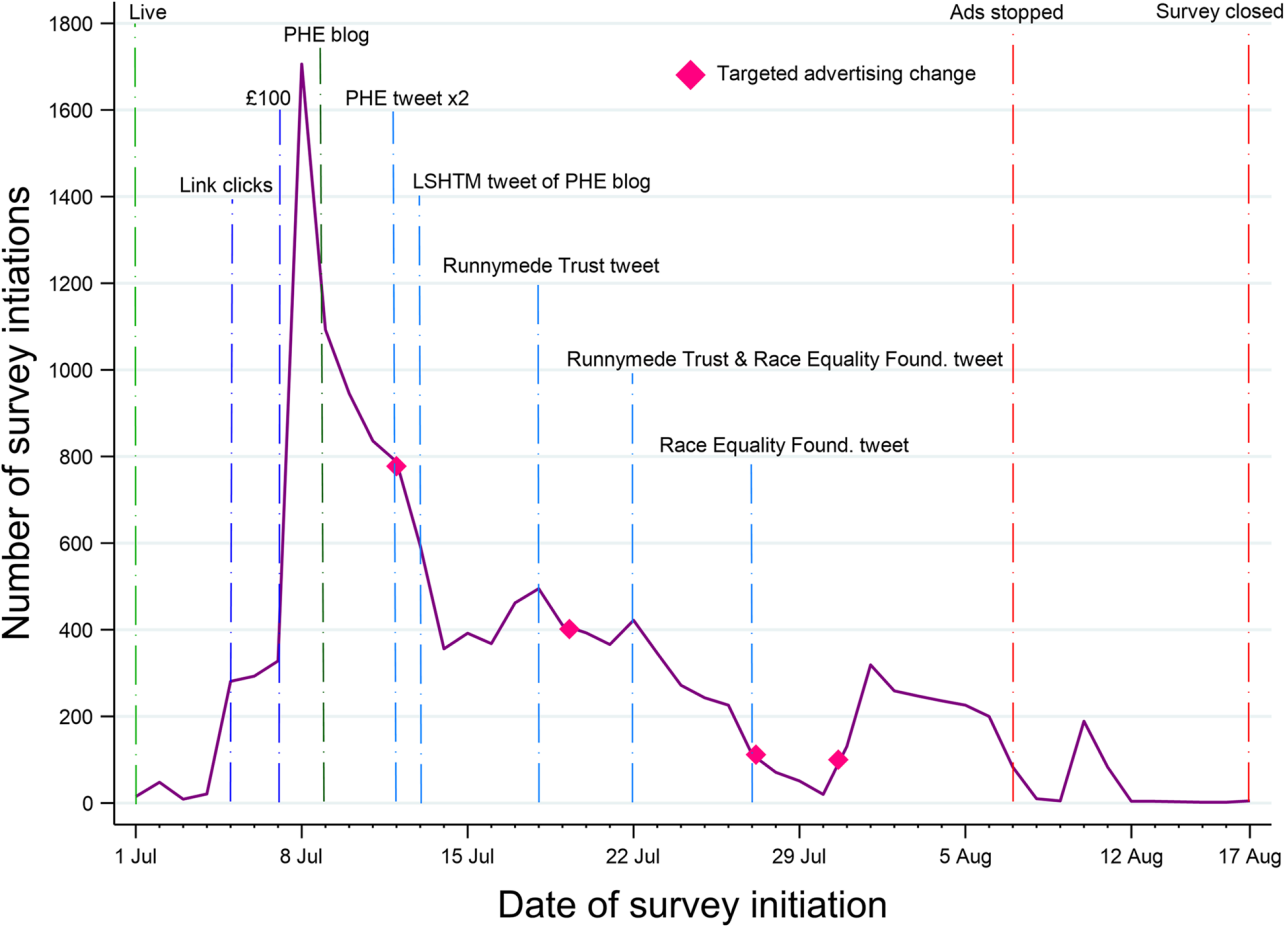
Annotated graph of strategies and response by day

The survey and the Facebook and Instagram advertisements went live on July 1 2021. Between this day and its close on 17 August (48 days), 13,962 people initiated the survey, with 11,578 fully completing it. The first few days saw less than 100 survey initiations per day, despite the wide reach of the advertisements. On day five, we changed the Facebook advertisement setting to ‘link clicks’ Table 1 provides details on the Facebook advertisement settings. On day seven, we increased the daily advertisement budget to £100 pounds per day and by day eight, we had approximately 1700 survey initiations- the largest daily number during the pilot. Figure 1 presents the number of survey initiations (includes completers and non-completers) by day, annotated with the recruitment strategies. The total spend on Facebook advertising was £4068.41, equating to £0.29 per survey initiation and £0.35 per survey completer.

**Table 1:** Facebook advertisement settings

Facebook setting	Explanation
Campaign objective	We selected ‘traffic’ to *‘Send people to a destination, such as a website, app, Facebook event or Messenger conversation’*
Campaign budget optimisation	*‘CBO may not spend your budget equally for each ad set. For example, if you have two active ad sets in your campaign, we might spend 90% of your budget on one ad set if that’s how we can get the overall best results.’* We turned this off as we did not want Facebook putting more budget behind ‘best performing’ ads, as we were looking for diversity and not just sheer volume of people
Automatic versus manual ad placements	We began with automatic placements whereby Facebook would decide where (e.g. main news feed, side bar, messenger) and on which platform (Facebook vs Instagram vs ‘audience network’) the adverts would be displayed We subsequently changed this to manual placements and removed: messenger, audience network, marketplace, video feeds, and group feed placements. This was after an initial review showing that the proportion of impressions converted to link clicks was particularly low on messenger
Optimisation for ad delivery	Initially we selected *‘Daily unique reach – We’ll deliver your ads to people up to once a day’* on the basis of preferring the advert to go to a wider audience fewer times rather than targeting those most likely to click on a link. However, after 10,000 impressions and no link clicks, we changed this setting to ‘*link clicks – We’ll deliver your ads to the people who are most likely to click on them’*

At the end of the first week, our review of the demographic spread of the sample necessitated a switch in the aim of our advertising from maximising recruitment to increasing the proportion of ethnic minority groups and the proportion who do not have a degree (or equivalent). During week two, we set the advertisements to be shown to Facebook and Instagram users who indicated on their profile that they had ‘up to some high school’. Also during week two, PHE published a blog on their website and tweeted twice and LSHTM tweeted about the PHE blog.

During week three, we continued with the ‘up to some high school’ setting. We also targeted the advertisements to be sent to users who indicated on their profile that they lived in a local authority with a high proportion of ethnic minority residents, which we identified though Office of National Statistics data. In week three, the Runnymede Trust also tweeted once. The proportion of Black British, Caribbean or African respondents recruited during week three (1.98%) was more than double the proportion recruited in week two (0.71%), however this still did not reach the proportion within the general population (4% in the 2011 Census). In week four, we again continued with the ‘up to some high school’ and local authority settings and added ‘high school leaver’. During week four, the Runnymede Trust tweeted again and the Race Equality Foundation tweeted once. Compared to the other recruitment weeks, week four saw the closest to proportional representation with regard to ethnicity, but still did not reach parity.

At the beginning of week five, we stopped the local authority targeting, continued with the education settings and also set the advertisements to be shown to users who work in industries that do not typically require a degree (administrative services, cleaning and maintenance services, sales, food and restaurants). Compared to week four, respondents in week five with a degree or equivalent fell 26%, from 83% in week four to 58% in week five. This, however, was still greater than the 46% in the 2011 Census with a degree. While there was greater proportional representation in education in week five, respondents who identified as White was the highest during this week, at 97%.

### Demographics of final sample

The characteristics of respondents within each recruitment week, and the cumulative proportion are presented in Table 2 alongside the characteristics of the population in England according to the 2011 Census. We had an over-representation of respondents who identified as being of White ethnicity (93.2%), and under-representation of respondents identifying as Asian/Asian British (2.2%) or Black British/Caribbean/African (1.5%). Almost 75% of our sample reported having a degree or equivalent qualification, substantially greater than the proportion in the general population with this level of education. The regional spread generally consistent with the English population statistics, with the exception of a slight over representation of those living in the Northeast and in London, and an under-representation of those living in the North West. Similarly, the age distribution of our sample was broadly reflective of the population of England, but with an under-representation of under-25-year-olds and an over-representation of respondents aged 25 to 44 years. 98.5% of respondents stated that they ‘think of themselves’ as female, 1.2% as non-binary, 0.11% as trans men, and 0.23% as ‘other’.

**Table 2:** Demographic characteristics of respondents

Demographic characteristic	Week 1		Week 2		Week 3		Week 4		Week 5		Week 6		Cumulative		*Census* *2011*
	%	n	%	n	%	n	%	n	%	n	%	n	%	n	
**Ethnicity**															
White	**93.92**	881	**94.43**	5,683	**92.19**	2,515	**89.26**	1,430	**96.56**	1,179	**89.4**	683	**93.19**	12,383	*83.50%*
Mixed or Multiple ethnic groups	**3.84**	36	**2.73**	164	**3.15**	86	**4.06**	65	**1.72**	21	**4.06**	31	**3.04**	404	*2.60%*
Asian or Asian British	**1.39**	13	**2.11**	127	**2.57**	70	**3.5**	56	**0.74**	9	**2.23**	17	**2.2**	292	*8.80%*
Black British, Caribbean or African	**0.75**	7	**0.71**	43	**1.98**	54	**3**	48	**0.9**	11	**4.06**	31	**1.49**	198	*4%*
Other ethnic group	**0.11**	1	**0.02**	1	**0.11**	3	**0.19**	3	**0.08**	1	**0.26**	2	**0.08**	11	*1.10%*
N		938		6,018		2,728		1,602		1,221		764		13,288	
**Has a degree or equivalent**															
No	**28.57**	262	**25.61**	1,516	**20.41**	549	**16.77**	265	**42.4**	508	**33.6**	252	**25.68**	3,356	*54%*
Yes	**71.43**	655	**74.39**	4,403	**79.59**	2,141	**83.23**	1,315	**57.6**	690	**66.4**	498	**74.32**	9,715	*46%*
N		917		5,919		2,690		1,580		1,198		750		13,071	
**Region**															
North East	**3.73**	34	**5.74**	330	**8.87**	233	**16.75**	263	**4.85**	56	**4.26**	31	**7.43**	948	*4.6%*
North West	**10.09**	92	**10.91**	628	**7.04**	185	**3.89**	61	**9.27**	107	**10.04**	73	**8.98**	1,146	*12.9%*
Yorkshire and Humber	**11.4**	104	**10.41**	599	**5.45**	143	**2.93**	46	**10.05**	116	**10.32**	75	**8.5**	1,084	*9.7%*
East Midlands	**7.89**	72	**8.79**	506	**7.5**	197	**4.71**	74	**11.96**	138	**7.43**	54	**8.16**	1,041	*8.4%*
West Midlands	**8.99**	82	**8.64**	497	**9.79**	257	**9.94**	156	**9.79**	113	**8.39**	61	**9.16**	1,169	*10.4%*
East	**10.64**	97	**11.85**	682	**9.1**	239	**3.82**	60	**11.79**	136	**11.14**	81	**10.17**	1,298	*10.7%*
London	**17.32**	158	**12.83**	738	**30.39**	798	**51.72**	812	**11.09**	128	**17.33**	126	**21.67**	2,765	*17.9%*
South East	**18.42**	168	**18.6**	1,070	**14.85**	390	**4.27**	67	**18.89**	218	**20.91**	152	**16.2**	2,066	*15.9%*
South West	**11.51**	105	**12.23**	704	**7.01**	184	**1.97**	31	**12.31**	142	**10.18**	74	**9.72**	1,240	*9.4%*
N		912		5,754		2,626		1,570		1,154		727		12,757	
**Age group**															
16–19	**14.11**	134	**5.28**	322	**3.92**	109	**1.47**	24	**0.73**	9	**1.3**	10	**4.51**	608	*8.3%*
20–24	**8.21**	78	**8.28**	505	**5.22**	145	**6.17**	101	**3.33**	41	**8.06**	62	**6.93**	934	*11.7%*
25–29	**18.95**	180	**14.03**	856	**12.95**	360	**14.67**	240	**11.95**	147	**22.37**	172	**14.52**	1,958	*12.9%*
30–34	**22.32**	212	**17.61**	1,074	**17.84**	496	**20.17**	330	**16.91**	208	**16.25**	125	**18.19**	2,452	*13.1%*
35–39	**13.16**	125	**14.36**	876	**16.22**	451	**17.79**	291	**13.74**	169	**13.13**	101	**14.94**	2,014	*13.0%*
40–44	**8.74**	83	**13.25**	808	**14.5**	403	**13.63**	223	**12.76**	157	**11.05**	85	**13.05**	1,760	*11.8%*
45–49	**6.63**	63	**11.74**	716	**12.3**	342	**11.74**	192	**14.07**	173	**10.79**	83	**11.65**	1,571	*12.9%*
50–55	**7.89**	75	**15.46**	943	**17.05**	474	**14.36**	235	**26.5**	326	**17.04**	131	**16.21**	2,185	*16.4%*
N		950		6,100		2,780		1,636		1,230		769		13,482	

We compared the demographic characteristics of those who went through the entire questionnaire and clicked submit, and those who did not. This showed a higher proportion of non-completers were aged 16–19 (7.9%) compared with completers (3.9%), and a lower proportion of non-completers had the highest level of education (degree or above) (67.9%) compared with completers (75.2%). Completers and non-completers had a similar profile in terms of ethnicity, gender, and region of residence.

### Item response among survey completers

Among respondents who completed the questionnaire fully (*n* = 11,578), the non-response to all but two key questions that all respondents were asked was less than 1%. The two exceptions were for the question about satisfaction with their sex life, where 14% did not respond and for the question about last vaginal intercourse, where 11% did not respond.

### Demographic characteristics of survey non-completers

We compared the demographic characteristics of those who went through the entire questionnaire and clicked submit, and those who did not. This showed a higher proportion of non-completers were aged 16–19 (7.9%) compared with completers (3.9%), and a lower proportion of non-completers had the highest level of education (degree or above) (67.9%) compared with completers (75.2%). Completers and non-completers had a similar profile in terms of ethnicity, gender, and region of residence.

### Reproductive health outcomes

Table 3 presents the proportions of the sample reporting selected health-related outcomes, the same proportions weighted to match the 2011 census for age and region distribution, and the equivalent prevalence estimates from 2010–12 Natsal-3 data (restricted to women aged 16–55 resident in England). The proportion of women in our sample who reported ever having an abortion and being pregnant in the last year were similar to Natsal-3 results. Abortion in the last year was under-reported in our sample (as in Natsal-3) when compared to government statistics. The most notable differences between our sample and Natsal-3 were our lower proportion of women who reported ever being pregnant (58.8% vs 69.6%), the higher proportion having ever experienced infertility (20.4% vs 13.0%), and the higher proportion of pregnancies in the last year being ‘planned’, as determined by the LMUP (76.5% vs 56.1%). The distribution of fertility intentions was broadly consistent with Natsal-3 results, with the exception of a slightly higher proportion reporting that they are currently trying to have (more) children (9.5% vs 6.9%). A lower proportion of respondents in our sample reported vaginal sex in the last 7 days (34.7% versus 46.4%). The effect of weighting the sample on age and region was minimal and did not consistently bring our estimates closer to those from Natsal-3.

**Table 3:** Reproductive health outcomes comparisons

**Outcome**	**RH-tracker survey**	**RH-tracker survey weighted**^g^	**Natsal-3** **(Women resident in England, aged 16–55)**
	**% (95% CI)**	**% (95% CI)**	**% (95% CI)**
**Ever been pregnant**	58.8 (57.9, 59.7)	55.5 (54.5, 56.5)	69.6 (68.2, 71.0)
**Ever had an abortion**	14.9 (14.3, 15.6)	14.3 (13.7, 15.0)	15.6 (14.5, 16.7)
**Pregnant in last year**^a^	13.9 (13.2, 14.6)	12.09 (11.4, 12.8)	14.0 (13.0, 15.1)
**(Live) birth in the last year**^a,^^e^	6.4 (5.9, 6.9)	5.4 (5.0, 5.9)	7.4 (6.6, 8.2)
**Ever infertility**^b^	20.4 (19.7, 21.2)	19.8 (19.0, 20.5)	13.0 (12.0, 14.1)
**Abortion in the last year**^a,^^f^	0.65 (0.5, 0.84)	0.74 (0.56, 0.98)	n/a
**Pregnancy planning (if pregnant in last year)**			
Unplanned	8.1 (6.4, 10.3)	10.5 (8.1, 13.5)	16.3 (13.0, 20.2)
Ambivalent	15.4 (13.0, 18.1)	15.9 (13.3, 18.8)	27.6 (23.5, 32.1)
Planned	76.5 (73.4, 79.4)	73.6 (70.0, 76.9)	56.1 (51.3, 60.9)
**Menopausal**^c^	41.8 (40.2, 43.4)	40.8 (39.1, 42.5)	45.14 (41.8, 48.5)
**Fertility intentions**^d^			
I would definitely like (more) children and I’m currently trying	9.5 (8.9, 10.1)	8.2 (7.7, 8.8)	6.9 (6.1, 7.8)
I would definitely like (more) children but I’m not currently trying	26.2 (25.4, 27.1)	26.8 (25.8, 27.8)	29.7 (28.3, 31.1)
I might (more) like children in the future—I’m not sure yet	20.3 (19.5, 21.2)	21 (20.1, 22.0)	19.3 (18.1, 20.5)
I would definitely not like (more) children	38.5 (37.5, 39.5)	38.6 (37.6, 39.7)	37.9 (36.2, 39.6)
I don’t know	5.5 (5.0, 5.9)	5.3 (4.9, 5.8)	6.3 (5.5, 7.2)
**Last occasion of vaginal sex**			
In the last 7 days	34.7 (33.8, 35.6)	33.9 (32.9, 34.8)	46.4 (44.9, 47.9)
Between 7 days and 4 weeks ago	24.7 (23.9, 25.5)	23.7 (22.9, 24.5)	21.5 (20.3, 22.8)
Between 4 weeks and 6 months ago	13 (12.4, 13.7)	12.3 (11.7, 13.0)	11.2 (10.2, 12.2)
Between 6 months and 1 year ago	5.5 (5.1, 5.9)	5.3 (4.9, 5.8)	5.1 (4.5, 5.8)
Between 1 and 5 years ago	10.9 (10.4, 11.5)	10.9 (10.3, 11.5)	6.1 (5.5, 6.8)
Longer than 5 years ago	5.2 (4.8, 5.7)	5.0 (4.6, 5.5)	3.6 (3.0, 4.2)
Never had vaginal intercourse	5.9 (5.5, 6.4)	8.9 (8.2, 9.6)	6.2 (5.6, 6.9)
**Self-rated health**			
Very bad	0.3 (0.2, 0.4)	0.3 (0.2, 0.4)	0.6 (0.4, 0.9)
Bad	3 (2.7, 3.3)	3.2 (2.9, 3.5)	2.7 (2.3, 3.2)
Fair	16.2 (15.6, 16.9)	16.8 (16.1, 17.5)	11.4 (10.4, 12.4)
Good	49.9 (49.1, 50.8)	50.1 (49.1, 51.0)	42.4 (40.9, 43.9)
Very good	30.6 (29.8, 31.4)	29.7 (28.9, 30.6)	42.9 (41.4, 44.5)

^a^under 45 years; ^b^Ever had a period of trying to get pregnant for more than 12 months and it hasn’t happened, among those who have ever had sex; ^c^not had a period for over a year, among those aged 45–55 years; ^d^under 50 years; ^e^5.75 per 100 women 15–44, 2019, England only. Source: https://www.ons.gov.uk/peoplepopulationandcommunity/birthsdeathsandmarriages/livebirths/bulletins/birthsummarytablesenglandandwales/2019/relateddata [accessed 22/09/2021]; ^f^1.83 per 100 women 15–44, 2020, England only. Source: https://www.gov.uk/government/statistics/abortion-statistics-for-england-and-wales-2020 [accessed 22/09/2021]; ^g^Data weighted to match 2011 Census profile for age group and region of residence

## Discussion

### Summary of main findings

This study sought to determine if a rapid online survey could achieve a proportionally representative sample compared to key demographics amongst women in England. The online social media recruitment strategies resulted in 13,962 people initiating our reproductive health questionnaire within 48 days. Of these, 11,578 respondents completed the questionnaire to the end and 2,384 exited the survey at various points. Daily monitoring and adjusting the Facebook advertisement settings to increase respondents from proportionally under-represented groups were modestly effective. During the week the advertisements were targeted by industry of employment, the proportion of respondents without a university degree or equivalent increased, however the proportion who identified as White increased to its highest point in the survey. The final sample did not achieve parity with England national statistics regarding ethnicity and education. Age group and region were closer to the national proportions, with younger people slightly under-represented. There was a greater than expected proportion of respondents reporting never having been pregnant, ever experiencing of infertility, and having a ‘planned’ pregnancy in the last year.

### Comparison with existing research

A study using a selection of Natsal-3 questions found that four non-probability online panel surveys were both less demographically representative of the general population and produced different key sexual behavioural estimates compared to Natsal-3 [15]. This is similar to what we found in our online non-probability sample, particularly with regard to ethnicity and education. The authors state that differences in the composition of the sample would contribute to some of the differences in sexual behaviour estimates between their online samples and Natsal-3. Similarly, we may attribute some of the differences in our sample to the recruitment strategy; for example, the higher proportion in our sample stating that they experienced infertility in the last year (20.4%, 95% CI 19.7–21.2) compared to Natsal-3 (13%, 95% CI 12.0–14.1) may be due to the age distribution, which was slightly older than the general population.

Another Natsal online panel survey (Natsal-COVID Wave 1) was conducted during the first UK national lockdown (for four months from 23 March 2020) to generate rapid estimates of the population’s sexual behaviours, needs and service use during the COVID-19 pandemic [10, 19]. The authors note the value in conducting such surveys, which include the ability to recruit a large national sample and to respond quickly to public health situations as needed, acknowledging the inability to produce reliable population estimates.

### Strengths and limitations

Our study has demonstrated that it is possible to obtain reproductive health information from a relatively large national sample in a short amount of time entirely online and for a relatively low cost per respondent. We have documented that efforts to achieve proportional representation in ethnicity and education remains a challenge through traditional social media platforms. Under-representation of people from ethnic minority groups or from those with lower educational qualifications has also been reported in other sexual health surveys using social media or online platforms for recruitment [20, 21]. A longer pilot period would have allowed deeper consultation with groups known to be under-represented in surveys. The survey was in English, with no other language options for completion. The under-representation of people aged 16–24 in our survey could have been due to young people increasingly choosing alternative social media platforms to Facebook. Future waves of the survey could expand recruitment to additional social media platforms or apps that are more popular with under-represented groups, however it is unlikely that the challenge of recruiting under-represented groups can be overcome by recruitment through social media alone. We cannot draw population estimates from our sample, however this was a recognised limitation from the beginning. Another limitation is that we do not have data, besides the Facebook settings, on people who were shown the advertisement but chose not to complete the survey- those that responded may have been more likely to have had a particularly poignant reproductive experience. In addition, the advertisement targeting relied on the users self-reporting information on their profile. Finally, we note the fundamental tension in using a platform designed for advertising to attempt to recruit a ‘random’ sample, whereby the adverts will be pushed to those most likely to engage with them based on an unknown and proprietary algorithm. Prior to the use of these platforms, considerations should weigh up the value of high numbers of respondents for a low cost versus representativeness.

### Implications

The finding that non-response to all but two key questions that all respondents were asked was less than 1%, supports the acceptability of both our survey questions and responses but also the acceptability of answering sensitive questions online. In Natsal-3 the majority of item non-response was under 2% [22]. It is not clear why non-response to last vaginal sex and sexual satisfaction questions (both Natsal questions) was high compared to the other more ‘sensitive’ questions (e.g. abortion in the last year). These two questions were located together in the questionnaire so one explanation could be a technical problem that we were unaware of.

While our sample provided estimates broadly comparable with those from Natsal-3 for several reproductive health outcomes, there were notable differences in the proportion reporting ever having been pregnant, experience of infertility, and the extent to which a pregnancy in the last year was ‘planned’. These differences may be explained by the different designs (our survey being a convenience non-probability survey versus Natsal being a probability survey) which led to our over-representation of highly educated women; Natsal-3 was conducted in 2010–12 so reproductive health outcomes may have changed over 10 years; and the COVID-19 pandemic may have an effect on reproductive health behaviours [19, 23], outcomes and experiences reported in our survey. Likewise, while the best data available, the Census data used is also 10 years old, so likely is not an accurate comparator.

Achieving the online questionnaire completions that we did involved constant monitoring and adjusting of the advertisement settings. While much of this work can be replicated for future waves of the survey, monitoring and adjustment will still be required. This will need to be carefully considered if the survey is administered outside a research setting.

The results of our strategy further underline the urgent need for data on the experience among ethnic minority groups and people without a degree. And more broadly, the results highlight the importance of collecting data that reflects the diverse range of reproductive health experience in England. While there is value in using a social media strategy to monitor women’s reproductive health and well-being at a national level over time, further research is needed with under- represented groups to understand and document how their beliefs and experiences influence their likelihood of responding to online surveys and to identify alternative means of ensuring their voices contribute to the improvement of reproductive health services and policies. A way forward for this tracker survey could be a hybrid of an online sample, with targeted, grassroots promotion of the survey by community organisations and leaders. This would involve engaging people who are digitally excluded plus additional specific inclusion health groups such as people who experience homelessness, sexual minority groups, people living with disability and vulnerable migrants. In addition, with use of translators for women whose primary language is not English, the provision of a paper-based questionnaire could be considered. While this would incur a larger financial investment than the online-only route, if effective, subsequent waves of the tracking survey could see a greater engagement and representation of these groups, particularly if they are done in regular intervals of one to two years.

Online surveys, like any activity conducted on the internet, are susceptible to being compromised by bots [24]- software that is intended to perform automated tasks to mimic a human. Because our pilot survey did not put in place protections to identify and prevent bots from completing it, it is not possible to determine if or to estimate the degree to which our data were affected. It is likely that cyberthreats such as bots are more of a risk, however, if there is an incentive to do so- e.g. a financial gain or a political incentive to promote misinformation and disinformation through the survey. Our pilot survey did not provide a financial incentive and was promoted as reproductive public health survey, rather than as a survey on a particular condition or experience that could have made it more of a target (such as an abortion survey). Future waves of the survey could limit this threat by including a CAPTCHA feature (Completely Automated Public Turing test to tell Computers and Humans Apart) to verify that a human being is attempting to complete it. To estimate the level of threat, we could use a website analytical tool to assess suspicious interaction with the survey website and identify non-human responses by inspecting the data for suspicious patterns, such as repeated responses.

## Conclusions

The social media strategy was successful in recruiting a relatively large sample within a short amount of time at a relatively low cost, however, it did not create a sample that was proportionally representative of the England population with regard to ethnicity and education level. We now need to collaborate with public, patient and community advocacy groups to understand and document the reasons why women from ethnic minority groups and women who have not completed a degree or equivalent (and other key groups whose experiences may have been missed by the strategy) do not respond to online reproductive health surveys such as ours. When this work is done, we then need to identify effective ways to meaningfully engage with and earn the trust of these women so that their reproductive experiences and needs are fully accounted for in our society and within health policy.

## Data Availability

In this paper, our aim is to report findings on our sampling strategy. The dataset generated from this pilot has not been fully analysed by the team and therefore is not publicly available at this stage. The dataset is available from the corresponding author on reasonable request.

## Abbreviations

LSHTM: London School of Hygiene & Tropical Medicine
Natsal: National Survey of Sexual Attitudes and Lifestyles
PHE: Public Health England
UK: United Kingdom

## References

[1] Morris DH, Jones ME, Schoemaker MJ, Ashworth A, Swerdlow AJ (2011). Secular trends in age at menarche in women in the UK born 1908–93: results from the Breakthrough Generations Study. Paediatr Perinat Epidemiol.

[2] Pokoradi AJ, Iversen L, Hannaford PC (2011). Factors associated with age of onset and type of menopause in a cohort of UK women. Am J Obstet Gynecol.

[3] Mishra G, Hardy R, Kuh D (2007). Are the effects of risk factors for timing of menopause modified by age? Results from a British birth cohort study. Menopause.

[4] Mishra GD, Cooper R, Kuh D (2010). A life course approach to reproductive health: theory and methods. Maturitas.

[5] Reproductive Health: World Health Organization; 2021. Available from: https://www.who.int/westernpacific/health-topics/reproductive-health.

[6] Sexual and reporductive health and rights: World Health Organization; [cited 2021]. Available from: https://www.who.int/teams/sexual-and-reproductive-health-and-research.

[7] Reproductive health: what women say. Information about the gaps in data and services in reproductive health and healthcare for women: Public Health England; 2018. Available from: https://www.gov.uk/government/publications/reproductive-health-what-women-say.

[8] Wellings K, Palmer MJ, Machiyama K, Slaymaker E (2019). Changes in, and factors associated with, frequency of sex in Britain: evidence from three national surveys of sexual attitudes and lifestyles (natsal). BMJ.

[9] Weatherburn P, Schmidt AJ, Hickson F, Reid D, Berg RC, Hospers HJ (2013). The European Men-Who-Have-Sex-With-Men Internet Survey (EMIS): Design and Methods. Sex Res Soc Policy.

[10] Dema E, Copas A, Clifton S, Conolly A, Blake M, Riddell J (2021). Methodology of Natsal-COVID Wave 1: a large, quasi-representative survey with qualitative follow-up measuring the impact of COVID-19 on sexual and reproductive health in Britain [version 1; peer review: 1 approved with reservations]. Wellcome Open Res.

[11] Mann S, Davison M, Logan L, Stanke C, Ratna N, Nardone A, et al. What do women say? Reproductive health is a public health issue. Public Health England, 2018.

[12] Internet users, UK: 2020: Office for National Statistics; 2021. Available from: https://www.ons.gov.uk/businessindustryandtrade/itandinternetindustry/bulletins/internetusers/2020.

[13] Social media usage in the United Kingdom (UK) - statistics & facts: Statista Research Department; 2021 [cited 2021 September 22 ]. Available from: https://www.statista.com/topics/3236/social-media-usage-in-the-uk/#topicHeader__wrapper.

[14] Arigo D, Pagoto S, Carter-Harris L, Lillie SE, Nebeker C (2018). Using social media for health research: Methodological and ethical considerations for recruitment and intervention delivery. Digital health.

[15] Erens B, Burkill S, Couper MP, Conrad F, Clifton S, Tanton C (2014). Nonprobability Web surveys to measure sexual behaviors and attitudes in the general population: a comparison with a probability sample interview survey. J Med Internet Res.

[16] Adults’ Media Use and Attitudes: Ofcom 2020 [updated 24 June 2020]. Available from: https://www.ofcom.org.uk/__data/assets/pdf_file/0031/196375/adults-media-use-and-attitudes-2020-report.pdf.

[17] Barrett G, Nolan EM, Gürtin ZB, Stephenson J, Hall JA (2020). London Measure of Unplanned Pregnancy and newer family forms: an update. J Epidemiol Community Health.

[18] Barrett G, Smith SC, Wellings K (2004). Conceptualisation, development, and evaluation of a measure of unplanned pregnancy. J Epidemiol Community Health.

[19] Dema E GJ, a Clifton S, Copas A, Tanton C, et al. Initial Impacts of COVID-19 on Sexual and Reproductive Health Service Use and Unmet Need in Britain: Findings from a Large, Quasi-Representative Survey (Natsal-COVID). Preprints with The Lancet. 2021.

[20] Wayal S, Reid D, Weatherburn P, Blomquist P, Fabiane S, Hughes G (2019). Association between knowledge, risk behaviours, and testing for sexually transmitted infections among men who have sex with men: findings from a large online survey in the United Kingdom. HIV Med.

[21] Coombe J, Kong FYS, Bittleston H, Williams H, Tomnay J, Vaisey A (2021). Love during lockdown: findings from an online survey examining the impact of COVID-19 on the sexual health of people living in Australia. Sex Transmitted Infections.

[22] Mitchell KR, Mercer CH, Ploubidis GB, Jones KG, Datta J, Field N (2013). Sexual function in Britain: findings from the third National Survey of Sexual Attitudes and Lifestyles (Natsal-3). Lancet.

[23] Mercer Cea. Early Impacts of the COVID-19 Pandemic on Sexual Behaviour in Britain: Findings From a Large, Quasi-Representative Survey (Natsal-COVID). Sex Transmitted Infections 97(Suppl 1) STI & HIV World Congress, 14–17 Jul 2021 A27 2021. 10.1136/sextrans-2021-055210 PMC8687784 34916335

[24] Pozzar R, Hammer MJ, Underhill-Blazey M, Wright AA, Tulsky JA, Hong F (2020). Threats of Bots and Other Bad Actors to Data Quality Following Research Participant Recruitment Through Social Media: Cross-Sectional Questionnaire. J Med Internet Res.

